# Catch Rates, Composition and Fish Size from Reefs Managed with Periodically-Harvested Closures

**DOI:** 10.1371/journal.pone.0073383

**Published:** 2013-09-16

**Authors:** Philippa Jane Cohen, Timothy J. Alexander

**Affiliations:** 1 ARC Centre of Excellence for Coral Reef Studies, James Cook University, Townsville, Australia; 2 WorldFish, Solomon Islands Office, Honiara, Solomon Islands; Aristotle University of Thessaloniki, Greece

## Abstract

Periodically-harvested closures are commonly employed within co-management frameworks to help manage small-scale, multi-species fisheries in the Indo-Pacific. Despite their widespread use, the benefits of periodic harvesting strategies for multi-species fisheries have, to date, been largely untested. We examine catch and effort data from four periodically-harvested reef areas and 55 continuously-fished reefs in Solomon Islands. We test the hypothesis that fishing in periodically-harvested closures would yield: (a) higher catch rates, (b) proportionally more short lived, fast growing, sedentary taxa, and (c) larger finfish and invertebrates, compared to catches from reefs continuously open to fishing. Our study showed that catch rates were significantly higher from periodically-harvested closures for gleaning of invertebrates, but not for line and spear fishing. The family level composition of catches did not vary significantly between open reefs and periodically-harvested closures. Fish captured from periodically-harvested closures were slightly larger, but *Trochus niloticus* were significantly smaller than those from continuously open reefs. In one case of intense and prolonged harvesting, gleaning catch rates significantly declined, suggesting invertebrate stocks were substantially depleted in the early stages of the open period. Our study suggests periodically-harvested closures can have some short term benefits via increasing harvesting efficiency. However, we did not find evidence that the strategy had substantially benefited multi-species fin-fisheries.

## Introduction

The challenge of sustainably managing small-scale fisheries in developing countries must be met to maintain food and livelihood benefits provided to millions of people [1]. Where alternative sources of income and dietary animal protein are limited, fisheries management must balance maintaining access to resources with avoiding or alleviating excessive fishing pressure. In many developing country contexts this has meant that permanent no-take reserves are not always a feasible option [2], [3], and that in many cases closures that are periodically harvested are preferred. In the Indo-Pacific, periodically-harvested closures have customary origins [4], [5], and emerge as important, or even primary management measures within many contemporary community-based and collaborative management arrangements (henceforth co-management) [6], [7].

In a centralised management context, rotational closures or periodically-harvested closures have been tested as a management strategy mainly for single-species invertebrate fisheries; scallops [8], [9], abalone [10], [11], lobster [12], sea urchins [13], [14] and coral [15]. Reported outcomes for invertebrate fisheries vary, suggesting that relative to strategies of continuous harvesting, periodic harvesting strategies can; (1) maintain population size, but will result in a decrease in yield [14], (2) maintain both population size and yield [13], [16], or (3) modestly improve biomass-per recruit and yield-per recruit, and decrease the risk of recruitment and growth overfishing [8]. Few studies have tested the effectiveness of co-managed periodically-harvested closures as a management strategy for fish, or for multi-species fisheries. Modelling of rotational closures suggests that for herbivorous fish, biomass and reef resilience can be improved [17], but when effort displacement is accounted for, net fisheries gains will be marginal [18]. Empirical field studies of multi-species fisheries suggest that where fishing is intense or prolonged, periodic harvesting strategies can lead to depletion of stocks that is more rapid or greater than recovery [19], [20]. Whereas, in other cases where fishing is light or only permitted for a short period of time, abundance and size of some fish can increase within the periodically-harvested area [21]–[23].

The success of periodically-harvested closures for managing fisheries broadly relies on growth and abundance increases within the area during periods of closure to be greater than or equal to levels of depletion during harvests. While gains in growth and abundance may lead to some secondary benefits, such as “spill-over” of adults and export of larvae to fisheries operating outside of the area, the marine reserve literature suggests that these benefits are slow to be realised, even where protection from fishing is permanent [24]–[26]. The recovery of exploited stocks and habitats when a fishing ground is closed depends on species demographics, site characteristics, the duration of the closure, hydrodynamics and larval supply [27], [28]. In the marine reserve literature, reported recovery rates vary from rapid and substantial increases in abundance as early as one to five years after the cessation of fishing [29], [30], to reports that relatively long periods of closure are required to build abundance and biomass of longer-lived, slower-growing fish species, and further that recovery rates can be dependent on unpredictable pulses of recruitment [25], [31]. Modelling of periodic harvesting suggests that relatively short cycles of closure and opening can build biomass sufficient to enhance yields of short-lived, fast-growing, sedentary species [10]. In general, periodic harvesting is predicted to be a more suitable strategy to maintain or enhance catches and stocks of sedentary, short-lived and fast-growing taxa (i.e., those of high rebound potential) than longer lived and slower growing species, or those with home ranges extending beyond the boundaries of the closure [32], [33].

In order for periodic-harvesting to enhance productivity in the long term, overall yield must be sustainable at greater levels than could be achieved by a continuous harvesting strategy. However, short term objectives of ‘saving up’ resources for specific times and enhancing catch efficiency, are also important objectives for communities and fishers in the Indo-Pacific [22], [34]–[36]. Elevated catch rates may result from increased abundance of fast growing taxa, or reduced flight initiation distance of finfish targeted by spear-fishers [37]. Whether short term improvements to catch efficiency correspond with sustainable or improved yields in the longer term is a pressing question for managers. For long-term objectives, patterns of depletion during harvesting events are equally important as recovery trajectories. Fishing patterns and resultant levels of depletion are driven by fisher behaviour, catchability of target taxa, gear selectivity and any restrictions placed on harvesting during the open period (and fishing patterns during closed periods if bans are not total or not fully complied with) [36]. In a multi-species fisheries context, exploitation patterns that result from a particular closure-harvesting cycle may lead to yield gains or stable populations of certain taxa, but might result in yield losses or depleted populations of others. To date there has been little research attention given to understanding the short-term and long-term consequences of periodic-harvesting for multi-species fisheries.

This paper examines the potential of periodically-harvested closures as a strategy to sustainably manage small-scale multi-species fisheries. In the context of Solomon Islands, we test whether the strategy can maintain or improve catch rates, and discuss the implications for longer term effects on yield. In four periodically-harvested closures we examine multi-species catch rates (catch per unit effort; CPUE), relative abundance of finfish and invertebrate families in catches, and compare the length of eight frequently-harvested finfish and one invertebrate species. We compare these observations with catches from the same group of fishers exploiting reefs that are continuously open to fishing. We test the hypotheses that when periodically-harvested closures are open to fishing: (1) catch rates are higher; (2) short lived, fast growing taxa are relatively more abundant; and, (3) finfish and invertebrates are larger, compared to harvests from reefs continuously open to fishing. In the case of one periodically-harvested closure, where adequate data were available (i.e., high frequency of trips), we also examine changes in CPUE and effort throughout the opening period to examine depletion.

## Methods

### Ethics

Research clearance, which included ethics clearance, was provided by the Minister for Education and Human Resource Development, Solomon Islands and by James Cook University, Australia under ethics approval number H3337. Interviewees gave their verbal consent to participate in the study and consent was noted on the interview transcript; if verbal consent was not given the interview did not proceed. Written consent was not sought because of low levels of literacy. The ethics committee approved the verbal consent process.

### Study location

Solomon Islands is a developing Pacific Island nation where coastal fisheries provide an important livelihood and the primary source of dietary animal protein in rural areas [38]. Communities, state government and non-government organisations (NGOs) are invested in co-management as the primary strategy to address small-scale fisheries management in Solomon Islands. Among a suite of strategies and management measures, most co-managed marine areas include some type of area closure, which in most cases is periodically-harvested [6].

**Figure 1:**
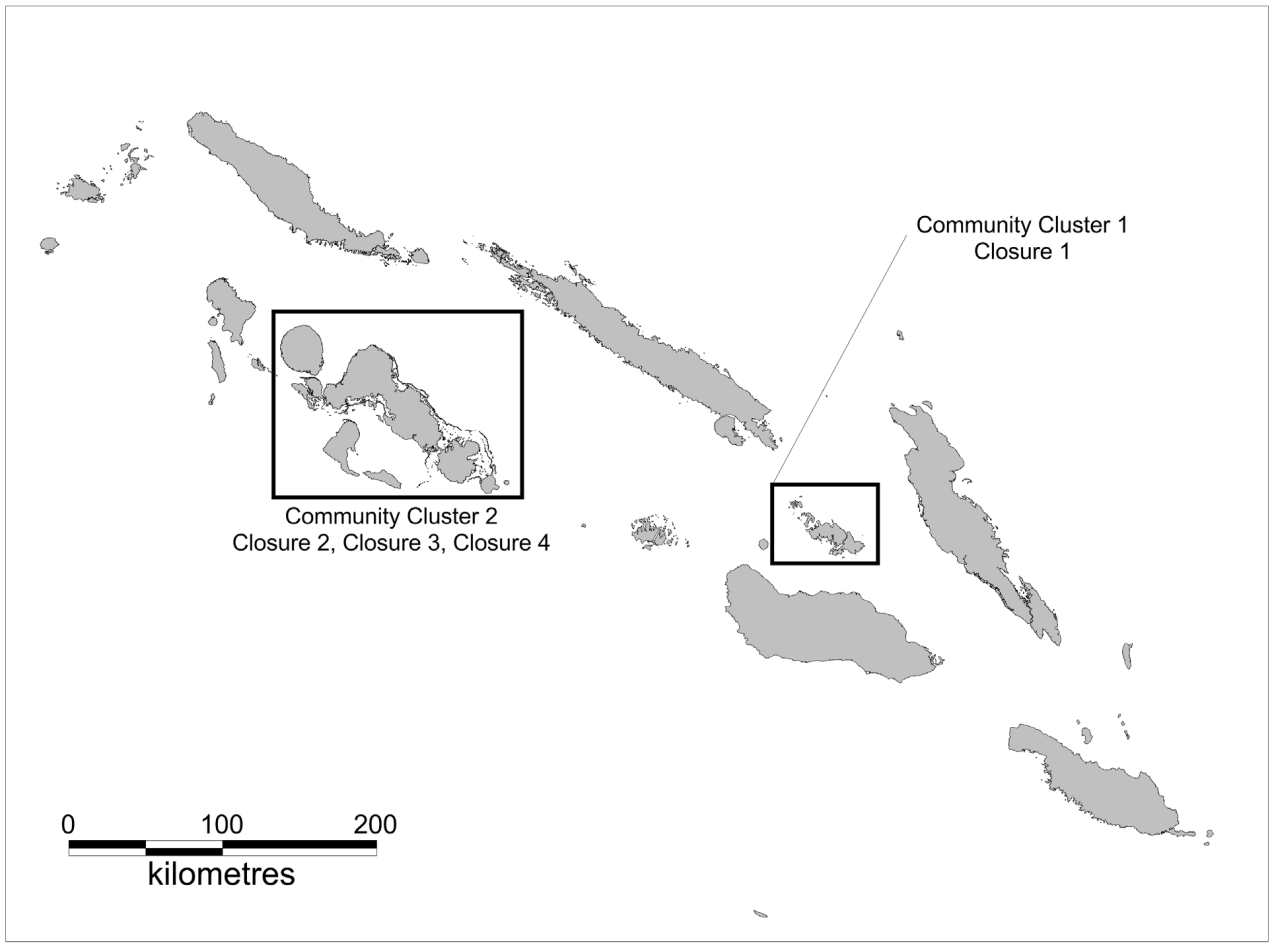
Map of study sites. The regions of Solomon Islands in which the two community clusters and four periodically-harvested closures were situated.

In this study, four periodically-harvested closures (henceforth referred to as Closures 1–4) were examined in two community clusters in Solomon Islands. Community names are not provided because of confidentiality arrangements, so they are referred to as community cluster one (CC1) and community cluster two (CC2; Fig. 1). Each community cluster consists of three separate, but geographically proximate, communities (i.e., between four and six kilometres from each other) who held fishing rights to the nearby fishing grounds, including the periodically-harvested closures. Fishers predominantly targeted reef areas, and also exploited pelagic and mangrove areas. While the majority of fishing was for subsistence purposes, there were also small-scale commercial fisheries in CC1 focused on trochus and reef fish, and only on trochus in CC2. Each of these communities had engaged in NGO-supported initiatives to develop co-management arrangements that included resource-use regulations and education, compliance and monitoring strategies.

As part of co-management arrangements, periodically-harvested closures were established over selected reefs. Reefs were generally selected by communities based on uncontested ownership and proximity to the village which allowed for easy monitoring and access. CC1 had one periodically-harvested closure, and CC2 had three, in which all extractive activities were banned during periods of closure. Periodically-harvested closures were all small (Closure 1: 0.04 km^2^, Closure 2: 0.63 km^2^, Closure 3: 0.03 km^2^ and Closure 4: 0.37 km^2^), accounting for less than five percent of the fished reef area (i.e., the total area of the 55 reefs observed to be used for fishing during the study period). Closure 1 at CC1 was established in 2005 and since then, until the harvesting event we observed, had reportedly been closed to all fishing activities, aside from the removal of coral that had been planted in the area. Closures 2, 3 and 4 at CC2 were established in 2008, and since that time had been predominantly closed each year for 11 months from January to November, and subjected to one month-long harvests every December.

### Sampling design and landing site sampling

We collected fishing trip and catch data from both community clusters. Although fishing took place in pelagic and mangrove areas, we only consider data from reef fishing grounds. Sampling coincided with community-planned openings of periodically-harvested closures (i.e., Closure 1 was harvested for 11 days in July 2011 and Closures 2, 3 and 4 were opened to harvesting for 31 days in December 2010). A trained observer recorded landings in each of the six communities during the full period of openings, and for at least two weeks during closures. Observers asked fishers to provide details of their fishing trip as soon as they returned to shore (n = 518 fishing trips), including: time of departure and time of return, method of transport (i.e., paddle canoe or boat with engine), number of fishers on the trip, gear(s) used, name of fishing ground(s), fished area description(s) (i.e., reef or other), and the harvesting strategy applied in the area (i.e., continuously open reef or periodically-harvested closure). Where method or fishing ground varied within a trip, these were specified and assigned to the appropriate fish or invertebrates within the catch. Trips were classified according to target type: finfish, invertebrate, mixed (i.e., finfish and invertebrates collected in the same trip) and ‘other’ (e.g., seaweed). Catch was weighed (i.e., total wet weight) using hanging scales (either a 10 kg/5 g digital scale or 22 kg/250 g analogue scale, depending on the size of the catch). Local nomenclature ([39], unpublished data) was used for counting and recording purposes. In 73 fishing trips, catches of the molluscs *Strombus luhuanus*, *Nerita polita* and *Polymesoda erosa* were too large to allow total enumeration of the catch. In these instances we sub-sampled the catch and extrapolated to the full sample.

In 86 fishing trips (i.e., of the total 518 trips observed), fishers were not immediately encountered at the landing location and their catch had already been cooked, consumed or sold. In these cases we used a ‘recall’ method to describe the landed catch; fishers were asked to provide the details of the fishing trip using the descriptors above. Fishers recalled the number and indicated the ‘average’ total length of each type of finfish or invertebrate using their hands; observers used a ruler to measure the size indicated. Recalled lengths were converted to weights using the standard expression W = aL^b^, and with length-weight (L-W) relationships from FishBase [40]. Before biomass estimation, we used length–length conversion factors from FishBase to change total length to fork length or standard length as the L-W relationship required. We preferentially selected L-W relationships derived from large samples and from the Indo-Pacific region, respectively. Where local nomenclature incorporated several species, we used the unweighted mean L-W coefficient to represent that grouping, and where it incorporated an entire family or genus, we used the L-W coefficient of the species we most frequently observed in catches. For species for which there was no L-W coefficient available we used that of another species of the same genus with similar morphology.

We excluded data from incomplete trip records, and from trolling trips due to low number of trips to periodically-harvested closures (Table 1). We also excluded data from trips using dynamite and nets because catches were distributed amongst many fishers, and so total catch weight from single netting or dynamiting events could not be reliably reconstructed. Note that the use of nets and dynamite were relatively infrequent, and their use in harvesting periodically-harvested areas was of similar frequency to their use on open reefs. Taking into account excluded data, a total of 191 fishing trips were recorded from the four periodically-harvested closures, and 327 trips from 55 reefs continuously open to fishing (henceforth ‘open reefs’), representing a total of 2 903 fisher hours (Table 2). We recorded 19 159 finfish and 19 043 invertebrates in total. A total of n = 213 (i.e., <1%) individual finfish or invertebrates were unidentified, and therefore excluded from the catch composition analysis.

**Table 1:** Data excluded from analysis.

Trip data excluded	n (trips)
Incomplete record (i.e. missing trip duration, catch weight, method)	33
Dynamite	19†
Nets	33†
Trolling	76

† The number of dynamite and netting trips refers to the number of fishers returning with catch from those events, and not the number of netting or dynamiting events.

**Table 2:** Sampling periods, and fishing trips and hours analysed in each location.

Reef area type	Days sampled	Fishing trips	
		# trips	# fishing hours
Closure 1	11	10	16
Closure 2	31	146	947
Closure 3	31	3	6
Closure 4	21	32	93
Open reefs CC1	23	130	765
Open reefs CC2	54	197	894

For length measurements the catch was photographed on a gridded sheet of plastic using a 12 megapixel camera. We measured and analysed length data for eight species of finfish (Table 3) and the invertebrate *Trochus niloticus* (trochus). These species were selected because they were numerically abundant in catches from both periodically-harvested closures and open reefs. Although abundant in catches, we did not measure the length of acanthurids as growth of adults is hard to detect (but acanthurids were included in catch weights) [41]. While data were originally recorded using local language names, images were used to identify fish to species level. Total lengths of finfish, and basal diameter of trochus, were determined through analysis of images using Image J [42]. In each image, all fish or trochus of interest were measured, and the data recorded against the corresponding trip details.

**Table 3:** Observed differences in length of fish and trochus caught from periodically-harvested closures compared to open reefs.

Species	Mean observed length difference (cm)	Mean % weight difference
*Lutjanus rufolineatus*	2.86 *	37.23
*Cephalopholis cyanostigma*	1.41	18.21
*Cephalopholis spiloparaea*	1.10	20.08
*Epinephelus merra*	0.82	11.93
*Variola albimarginata*	0.80	9.06
*Balistapus undulatus*	0.50	9.25
*Cephalopholis urodeta*	−0.49	−8.80
*Melichthys vidua*	−0.25	−4.82
*Trochus niloticus*	−0.34 *	−20.79

Mean % weight difference is calculated from the mean average length difference and using the standard expression W = aL^b^.

* indicates lengths were significantly different between periodically-harvested closures and open reefs.

### Data standardisation

Prior to pooling data collected by the recall and direct observation methods, we used a two sample t-test to determine whether the data collection methods varied in terms of trip duration and catch weight. Trip duration and catch weight were square root transformed to improve normality which was assessed by inspecting residual plots. There was no significant difference (t = 0.03, df = 516, p = 0.787) between the average trip duration for those trips observed directly (313±13 minutes, n = 432), and those recorded using the recall method (314±28 minutes, n = 86). As we were interested in the comparability of the recall and direct observation methods for estimating catch weight, we excluded trips where nothing was caught. Using a Welch modified two-sample t-test to account for unequal variances, we found there was a significant difference (t = −3.14, df = 95, p = 0.002) between catch weight from trips observed directly (4.03 kg±0.30, n = 423), and those collected using the recall method (5.09 kg±1.92, n = 86). There was no systematic bias in the use of the recall method for collecting data from any fishing method or any harvesting strategy (i.e., continuous or periodic). Accordingly, we adjusted catch weights from the recall method with a correction factor of 0.8. Subsequent analyses were run with and without data collected with the recall method, and this did not vary the main findings.

Catch rate (i.e., CPUE) was calculated for each trip and expressed in kilograms per fisher hour. CPUE provides a proportional index of abundance where catchability is constant. However, a range of factors can influence catchability and either accentuate or dampen changes in catch rates relative to actual fish abundances [43]–[45]. We minimise most factors as our sampling was geographically and temporally discrete, stratified by gear type, and observations suggested fisher skill level was randomly distributed within sampling times and locations. We later discuss the potential influences of fish behavioural responses and target switching (i.e., when fishers change their target taxa) on the relationship between CPUE and abundance. The design of our study therefore allows us to discount many of the confounding factors usually attributed to CPUE, while the sensitivity of the metric to changes in abundance and capture effort make it a particularly appropriate index for use in this context.

To standardize catch rate for reef fishing we estimated and removed travel time to and from reefs, so that the time component of effort accounted for active fishing only. In each community, we asked experienced fishers to estimate travelling times (i.e., via canoe as this was the only boat type used for fishing on reefs) to fishing grounds they were familiar with. We calculated distances to these fishing grounds using MapInfo 11.0 and then calculated a median paddling speed (9 minutes km^−1^) to infer travelling times for all other reef fishing grounds. Subsequently, according to the distance between the fishing ground and landing location of each trip, we determined actual time spent fishing by subtracting paddling times from total trip time.

### Data analysis

To compare the difference in CPUE between harvesting strategies (i.e., periodic versus continuous harvesting) we used a linear mixed effects model [46] using S+ (version 8.2). Harvesting strategy and fishing method were treated as fixed factors, and we tested for interaction effects (i.e., harvesting strategy x fishing method). The model contained two random factors; region (i.e., CC1 or CC2) and fishing ground (i.e., the 55 open reefs and the four periodically-harvested reefs) which was nested within region. CPUE data were strongly skewed; a reciprocal transformation (i.e., 2 - (1/CPUE+0.5) improved normality. We examined residual plots to confirm data were normally distributed, and equal variances were confirmed using Levene's test.

In Closure 2 there were sufficient trips through the cycle of opening to allow an analysis of trends in CPUE from the commencement of harvesting until the end. The comparison of CPUE from open reefs was restricted to only those reefs in the same region as Closure 2 (i.e., CC2 open reefs). CPUE averaged over each week of the opening period were initially visually inspected because the sporadic timing of fishing trips, and uneven distribution of effort between open reefs and Closure 2, meant that it was difficult to conduct formal statistics of CPUE trends through time. Based on this visual inspection and the relatively low frequency of fishing trips in the later stages (i.e., final three weeks) of the harvesting period, we categorised trips into those occurring in the early (i.e., first seven days) or the later (i.e., final 24 days) stages of the periodic harvest. We ran a two-way ANOVA with harvesting strategy-time (i.e., ‘periodic harvest-early’, ‘periodic harvest-later’ or ‘open reef’), and gear as independent variables. We used Tukeys post-hoc tests to determine where differences lay.

All catch composition analyses were conducted in PRIMER [47] following the methods described in Clarke and Warwick [48]. Catch composition data were first standardised by effort, dividing the total number of individual fish and/or invertebrates caught at each particular fishing location by the total number of fisher hours sampled at that location (summarised in Table 2). A few families (e.g. Strombidae and Acanthuridae) were particularly abundant in catches. Therefore data were square root transformed so as to increase the sensitivity to detect differences driven by families of intermediate abundance. Non-metric multidimensional scaling (MDS) based on Bray-Curtis similarity measures was used to examine variability in catch composition between sites. Due to the high stress of the two dimensional MDS, we also consulted the three dimensional version of the plot to confirm that patterns were not being misrepresented in two dimensions. ANOSIM was used to test whether the catch composition was significantly different between periodically-harvested closures and open reefs, and SIMPER analysis identified the families important in driving the trends. We analysed all fishing methods together, and also examined results from analyses conducted separately for gleaning for invertebrates, spear fishing for finfish, and line fishing for finfish. Where periodic harvests spanned several weeks (i.e., Closures 2 and 4), we visually examined catch composition through time. We characterised fish families in the catches as having a low, medium or high potential to recover from fishing (referred to henceforth as ‘rebound potential’) based on species dominant in catches and the species-specific index for resilience to fishing reported by FishBase and SeaLifeBase [40],[49].

We restricted analysis of fish and trochus size to Closures 2, 3, and 4, in comparison to open reefs in CC2; we did not examine Closure 1 due to the few replicates of most species in catches relative to species in catches from open reefs in the same region. Length data for the eight finfish species (n = 1 216) and for trochus (n = 312) were analysed separately. Data were log-transformed to improve normality, and variances tested with Levene's test were found to be equal. We used a one-way ANOVA to examine the effect of periodic versus continuous harvesting strategies on the length of trochus, and each of the eight finfish species. Finally, length-weight relationships were used to calculate the difference in weight of average size fish caught on open reefs compared to average size fish from periodically-harvested closures. Species-specific growth parameters were retrieved for finfish from FishBase [40], and for trochus from Nash et al. [50]. Where parameters were not available for a particular species, we used those of the family [40].

## Results

### Catch rates

**Figure 2:**
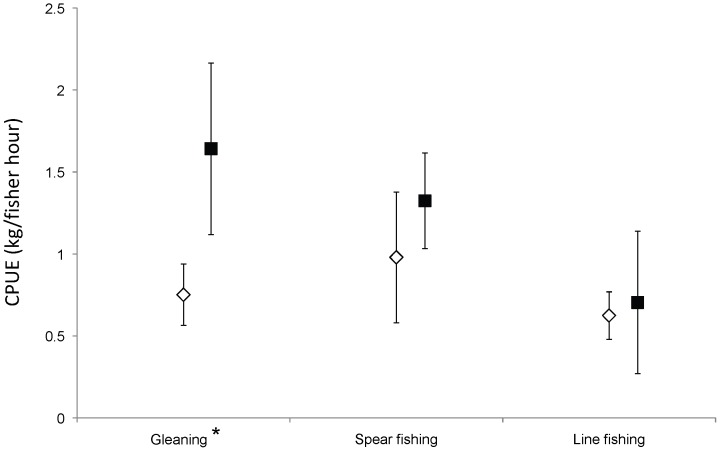
Catch rates from periodically-harvested closures and open reefs, by fishing method. Catch rates (untransformed CPUE) from commonly used methods of harvesting in periodically-harvested closures (closed symbols) and continuously open reefs (open symbols). Error bars indicate 95% confidence intervals. * indicates a significant difference at α = 0.05.

Catch rates were significantly higher from periodically-harvested closures than from reefs continuously open to fishing (F_1, 455_ = 9.93, P<0.01), yet this effect varied significantly between fishing methods (interaction between harvesting strategy and fishing method F_3, 455_ = 3.03, P<0.05; Table 4). Due to the significant interaction effect, we re-ran the analysis for each fishing method separately. Catch rates from gleaning were twice as high from periodically-harvested closures as from reefs continuously open to fishing (F_1,91_ = 2.74, P<0.01), whereas catch rates from spear fishing and line fishing did not differ significantly, but the trend was the same (Table 4; Fig. 2).

**Table 4:** (A) CPUE data in linear mixed-effects models with harvesting strategy and fishing method as fixed factors, region and fishing grounds as random factors (B) Separate models for each fishing method.

**(A) Full model**						
	df	F-value	p-value			
(Intercept)	1	156.13	<0.001	***		
Harvesting strategy	1	9.93	0.002	**		
Fishing method	2	18.13	<0.001	***		
Harvesting strategy x Fishing method	2	3.03	0.049	*		
**(B) Fishing methods analysed separately**						
Gleaning	Value	Std.Error	df	t-value	p-value	
(Intercept)	1.00	0.06	91	17.6	<0.001	***
Harvesting strategy	0.32	0.12	91	2.7	0.007	**
Line-fishing	Value	Std.Error	df	t-value	p-value	
(Intercept)	0.84	0.04	160	22.7	<0.001	***
mgt.broad	−0.01	0.09	160	−0.1	0.923	
						
Spearing	Value	Std.Error	df	t-value	p-value	
(Intercept)	1.01	0.13	167	7.5	<0.001	***
mgt.broad	0.13	0.12	167	1.1	0.254	

**Figure 3:**
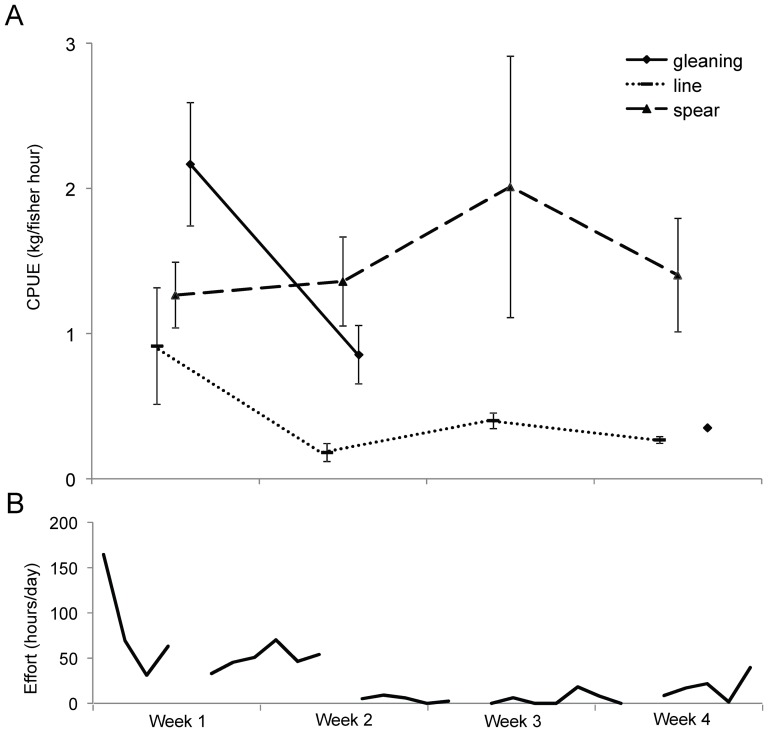
CPUE and effort throughout a periodic harvest. Each week of the one month harvest period of Closure 2 (A) Mean weekly CPUE (± SE) (B) Daily fishing effort (total fisher hours per day) applied throughout the harvest period. Gaps in effort data represent Sundays when no fishing took place for social reasons.

In Closure 2 there were sufficient trips through the cycle of opening to examine whether catch rates declined during the harvest. Visual inspection of the data (Fig. 3A) indicated that relatively high catch rates for gleaning and line fishing declined after the first week of harvesting, whereas spear fishing catch rates were variable throughout the harvest period. Total fishing effort applied throughout the opening period (Fig. 3B) was particularly high on the first day of opening, remained high for the first 11 days, then declined and remained relatively low through the remaining 20 days of the open period. CPUE varied significantly between fishing methods (F_2, 344_ = 19.78, P<0.001), but did not vary between harvesting strategy-times; i.e., early in the periodic harvest, later in the periodic harvest and harvesting from open reefs (F_2, 344_ = 1.54, P = 0.216). Due to a near-significant interaction between harvesting strategy-time and fishing method (F_4, 344_ = 2.11, P = 0.079), we examined each of the three methods separately. CPUE for gleaning, but not for line fishing and spear fishing, significantly varied between harvesting strategy-time (Table 5). Tukey post-hoc tests revealed that for gleaning, CPUE was significantly higher in the early stages of periodic harvesting compared to open reefs, but not in the later stages.

**Table 5:** Mean CPUE and ANOVA results of CPUE in the early and the late stages of the periodic harvest of Closure 2, and CPUE from open reefs in the same region (i.e. CC2).

	Continuously open reef		Early periodic harvest		Later periodic harvest		ANOVA	
	Mean ± SE	n	Mean ± SE	n	Mean ± SE	n	F	P
Spear fishing	1.42±0.44	33	1.27±0.22	28	1.45±0.24	53	0.34	0.714
Line fishing	0.67±0.09	142	0.91±0.40	15	0.28±0.04	11	1.02	0.363
Gleaning	1.03±0.24	22	2.17±0.43	25	0.80±0.19	9	3.71	**0.031** *

CPUE is catch per unit effort measured in kilograms per fisher hour.

* indicates statistical significance at the level α = 0.05.

### Catch composition

**Figure 4:**
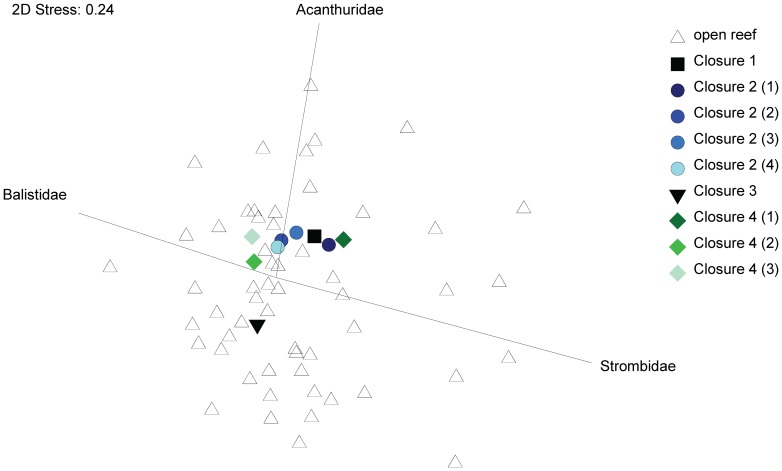
Distribution of sites based on catch composition. Non-metric multi-dimensional scaling plot of counts from families comprising catches from all fishing methods, from individual open reefs (open symbols) and periodically-harvested closures (closed symbols), with overlaid vectors of three families contributing most to observed variation between reefs. For Closures 2 and 4 (i.e. where periodic harvests spanned several weeks), the number in brackets indicates the week of harvesting (i.e. first to fourth week).

We recorded the capture of 36 families of finfish, 15 families of invertebrates, and five other families. We examined family level composition of catches from all methods combined, and then separately for spear fishing and line-fishing for finfish, and gleaning for invertebrates in the four periodically-harvested closures and 55 open reefs. Considering reefs as replicates, MDS plots suggested no clear differentiation between catches from open reefs and periodically-harvested closures for all methods combined (Fig. 4), or for each of the three fishing methods.

ANOSIM results confirmed that family level composition of catches from periodically-harvested closures and open reefs did not vary significantly for all three fishing methods combined (*R* = −0.141, *p* = 0.79), or for gleaning (*R* = −0.023, *p* = 0.531), spear fishing (*R* = −0.248, *p* = 0.60), or line fishing (*R* = 0.046, *p* = 0.39) when analysed separately. SIMPER results indicated that any dissimilarity that did occur between composition of catches was driven mainly by relatively higher abundances of Strombidae (for gleaning), Acanthuridae (for spear fishing), and Balistidae (for line fishing) in catches from periodically-harvested closures (Table 6). These three families had intermediate to high potential to rebound from fisheries exploitation [40], [49].

**Table 6:** Results from SIMPER analysis for the families that contribute most to dissimilarity in composition of catches from each fishing method from open reefs compared to periodically-harvested closures.

		Average number of individuals per fisher hour			
Fishing method	Family	Open reefs	Periodically-harvested closures	% contribution to dissimilarity	Rebound potential†
Gleaning	Strombidae	1.77	5.15	45.85	high
Spear fishing	Acanthuridae	1.21	2.46	15.98	intermediate-high
Line fishing	Balistidae	0.10	1.00	16.91	intermediate

† Rebound potential from fisheries exploitation is based on the life history characteristics of each family [40], [49].

### Fish and trochus lengths

**Figure 5:**
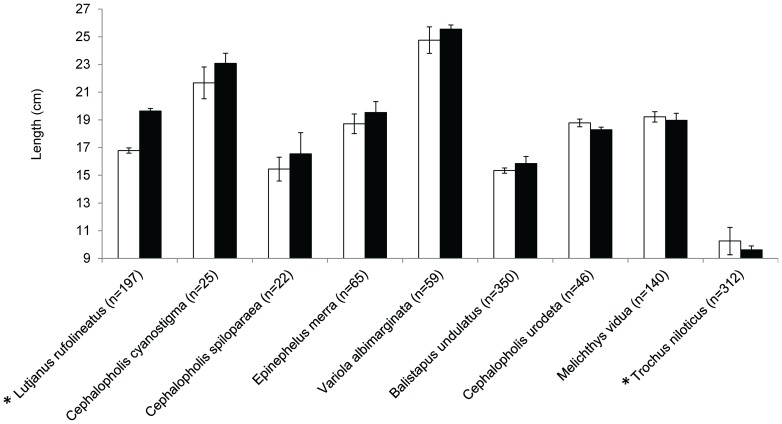
Finfish and trochus lengths. Average lengths of finfish and trochus harvested from open reefs (white bars) and periodically-harvested closures (black bars). Error bars indicate standard error. * indicates a significant difference between length of fish or trochus caught from periodically-harvested closures compared with open reefs.

One way ANOVAs for each species identified that only *Lutjanus rufolineatus* was significantly larger (F_1, 195_ = 7.97, P = 0.005) from periodically-harvested closures compared to fish of the same species caught from open reefs (Figure 5). Six of eight finfish species were observed to be larger from periodically-harvested closures and observed differences in length translated to an average difference of 11.5% in weight (Table 3). Trochus were significantly smaller in catches from periodically-harvested closures compared to those harvested from open reefs (F_1, 310_ = 5.9425, P = 0.015). The observed difference in length translated to a −21% difference in weight per individual.

## Discussion

Periodically-harvested closures are an important component of co-management in the Indo-Pacific [6], [51]. Communities and their partner agencies expect that periodically-harvested closures will deliver short-term gains by improving catch rates, and long-term benefits by improving or sustaining yields. However, empirical evidence of these benefits, and the circumstances in which they can be realised, has been lacking [36]. During the periodic harvesting events we observed, catch rates for gleaning of invertebrates were higher than those from continuously-open reefs. Catch rates for fish were not elevated, but fish caught from periodically-harvested closures were slightly larger than those caught from open reefs. While enhanced catch rates and larger fish are important short-term benefits for fishers, the long-term benefits and sustainability of the strategy will depend on the frequency and levels of exploitation during periodic harvests. The relatively intense harvesting we observed probably caused localised depletion of sedentary invertebrate stocks. Our observation of elevated catch rates for gleaning (but not for other methods) supports the prediction that periodic harvesting is better suited for managing fast growing, short-lived, sedentary or sessile taxa. We did not find evidence that the strategy had been beneficial for the management of multi-species fin-fisheries.

### Are catch rates improved in periodically-harvested closures?

By employing a periodic harvesting strategy, fishers potentially benefit from increases in growth and abundance accrued during periods of closure. From an ecological perspective, the extent of recovery during closed periods will depend on the status of the stock and condition of the habitat when the closure commences, the life history characteristics and scales of movement of target species, alongside patterns of larval dispersal and recruitment that are influenced by hydrodynamics and stock status in surrounding areas [28]. Recovery will also depend on harvesting dynamics including gear selectivity and habitat impact and the duration, frequency, and intensity of harvests. An important social objective of implementing periodically-harvested closures is that resources are stockpiled for harvesting in times of high demand [21], [52]. This goal may be valued more by communities than net increases in productivity and may therefore outweigh the cost of reduced access to fishing grounds during periods of closure. In line with this goal, the harvesting events we studied were initiated when communities had elevated social (e.g., celebratory feasts) and economic (e.g., fundraising) needs for marine resources. The higher catch rates we observed for gleaning suggest that in some situations, periodically-harvested closures can allow fishers to harvest more efficiently to address their immediate and elevated needs for marine produce. However, in situations where populations are critically low, even fast growing, highly fecund species, and particularly sedentary taxa, may not recover during periods of closure due to reduced fertilisation success (i.e., the Allee effect; [53]–[55]). Where elevated catch rates are achieved, this may or may not correspond with longer term objectives of maintaining or improving yield – the characteristics of harvesting will also mediate long term outcomes.

Improved catch rates were not evident for line or spear fishing. This suggests that closure periods were short relative to the time required for fish to rebuild from previous harvesting events. Even for sedentary invertebrate stocks, modelling of optimal harvesting cycles and harvesting levels suggests the benefits of periodic harvesting may only be modest [8]. Further, harvesting during closure periods (e.g., due to infringements) may mean that benefits for fishers harvesting during the scheduled periods of opening are reduced, or not realised at all. In fact, in a parallel study of the same four closures it was found that there were low levels of harvesting (i.e., through both non-compliance, and a limited number of approved harvest events) during the intended periods of closure [56]. In addition to changes in biomass and abundance, patterns of fishing and protection can also influence catch rates via changes in fish behaviour i.e., fish become less fearful of fishers after periods of protection [37], [57]. In particular, acanthurids tend to display this response [37], [57], [58] and our observation of a relatively high composition of acanthurids in spear fishing catches from periodically-harvested closures may reflect this effect. In some cases, short-term elevation of catchability is an explicit objective of implementing periodically-harvested closures [22]. However, in these cases we did not observe significant increases in catch rates of fish from periodically-harvested areas that could be attributed to substantial behavioural changes, or increases in abundance or biomass.

Pulses of fishing can be intense when closures are opened, particularly where participation in harvesting is unrestricted, governance institutions are weak, fishers anticipate improved catch rates, or when demand and needs are high [20], [33], [59]. During the harvesting events we observed, average daily effort was between four and 60 times higher than effort on nearby open reefs [56]. Declines in catch rates from gleaning between the early and later stages of the harvesting of Closure 2 suggest that harvesting led to substantial localised depletion of invertebrates. A similar decline in catch rates was apparent, but not significant, for line fishing. Evidence of invertebrate depletion was also provided by anecdotal reports from fishers, and a decline in effort (for gleaning in particular) throughout the periodic harvest (Fig. 3B). The significantly higher catch rates for gleaning in periodically-harvested closures overall were therefore likely due to a few days of good catches at the commencement of openings, rather than consistently good catch rates throughout. These trends are probably accentuated for gleaning where conspicuous individuals are quickly removed, and subsequently more time must be spent locating and harvesting remaining cryptic individuals. Observations of stock depletion from other periodically-harvested closures in the Indo-Pacific region vary, due in part to differing opening and closure cycles. In Papua New Guinea for example, a one day harvest caused no significant impact on biomass [21]. Yet in Hawaii, declines in abundance of target-species indicated that the one to two year closure periods were too short for growth and reproduction to compensate for depletion during the one to two year-long openings [19]. Where depletion of stocks during opening periods exceeds recovery, such as observed in Hawaii, periodically-harvested closures will not meet long-term sustainability objectives. In situations where fishing can be intense, restrictions on frequency, duration and intensity of periodic harvests will likely be necessary to realise long-term fisheries goals.

Applying a range of management measures may increase the likelihood of positive fisheries outcomes from co-management [60]. The co-management arrangements we studied also included a range of other fisheries regulations including size limits, gear restrictions etc., however few of these were implemented and enforced in practice [56]. Similarly, the use of other fisheries regulations to complement periodically-harvested closures were infrequently reported in cases from across the Indo-Pacific [36]. In high fishing pressure contexts, and where harvesting is effectively unrestricted, depletion can be substantial when marine closures are opened to fishing (e.g., [31], [61]). Additionally, periodically-harvested closures in the Indo-Pacific are often very small [36]; the areas we studied were all less than 0.1 km^2^, and accounted for less than 5% of all reef area fished by communities. Even where periodically-harvested closures can improve fisheries within their boundaries, given escalating pressures from growing populations and developing markets, a diversity of strategies will be required to effectively manage fisheries in the spaces between closures [62], [63]. However, periodically-harvested closures may prove to be useful foundations to improve understandings of fisheries limits and to build capacity to monitor and enforce local controls on resource use.

### Does the composition of catch vary from periodically-harvested closures?

Although frequently applied to manage multi-species fisheries, few studies have critically compared the outcomes of periodically-harvested closures for different taxa (but see [22], [23]). We observed no significant differences in the familial composition of catches from continuously harvested reefs or from periodically-harvested reefs. The small amount of dissimilarity that did exist between catches from the two harvesting strategies was mainly driven by relatively higher counts of invertebrates or fish from families with intermediate or high rebound potential. In the two periodically-harvested closures where there were sufficient data to study catch composition through time, strombids were somewhat more abundant in catches from newly opened areas compared to catches from open reefs. As harvesting continued, we observed a weak signal of catches becoming more similar to those from open reefs as the relative abundance of strombids in catches decreased (Fig. 4). This further supports our observation that gleaners profit most from increases of faster growing invertebrates, but that these benefits are reaped mainly in the early stages of the harvest. The lack of a significant change in catch composition from each method through time, or between harvesting strategies, also allows us to assume that fishers did not systematically change targeted species over the course of the study which could have affected CPUE relative to abundance.

While our observations of elevated catch rates provide some evidence that stocks of short lived, fast growing taxa had built during periods of closure and may be suited to management with periodically-harvested closures, other cases from across the Indo-Pacific demonstrate a variety of outcomes. Trochus have a high rebound potential, but were observed at relatively low abundance in a closure in Vanuatu, and were therefore considered to be vulnerable to the periodic harvesting strategy applied there [23]. In another case in Solomon Islands, trochus catches had declined, and populations were relatively low where periodically-harvested closures were employed and harvests were only minimally restricted [64], [65]. By contrast, higher, sustained abundances of trochus in Cook Islands were attributed partly to management with a combination of scientifically informed size limits, quotas and harvesting cycles [50]. Some observations also indicate that periodic harvesting can benefit species vulnerable to exploitation (such as larger, longer-lived taxa) in circumstances where fishing pressure is low during harvest periods, or where total effort has been reduced because of the decreased opportunity to harvest. For example, in Vanuatu relatively higher abundance and biomass of tridacnid clams and fish with vulnerable life histories were observed inside periodically-harvested closures [23], and in Papua New Guinea there were relatively higher abundances of families of long-lived fish with long population doubling times [22]. In some cases, the preferential selection of a productive fishing ground for periodic closure may enhance the differences observed in abundance or catches between continuously-fished areas and periodically-harvested closures. Population dynamics within an area are not only influenced by the direct effects of closure and periodic harvesting; migration, external recruitment and fishing patterns in surrounding areas are also important drivers, particularly when closures are small and for species with home ranges that extend beyond the boundaries of the closure or that have highly dispersing larvae [28], [31]. Fisher and community expectations of what might be achieved for their fisheries by implementing periodic harvesting strategies should be tempered by the variability of these outcomes.

### Are fish larger from periodically-harvested closures?

Temporarily removing or reducing fishing pressure in an area may enhance yield per recruit by permitting continued growth and accumulation of larger individuals [16], [66]. We observed that individuals of some finfish species taken from periodically-harvested closures were slightly larger than those from open reefs. *Lutjanus rufolineatus* was the only species that was significantly larger, yet had a relatively moderate (i.e. fourth highest) growth rate compared to the other seven species we analysed. This highlights that average fish size on any particular reef is not simply a function of growth rate, but also influenced by historical fishing patterns [67]. As most fishing methods are size selective towards large individuals [68], ceasing fishing in an area can change the size spectra of fish communities so that large fish are relatively more abundant. This effect was observed in Papua New Guinea, where fish on reefs that had been harvested two to three times per year were larger on average than fish in continuously fished areas [21]. The periodically-harvested closures where fish were captured for our study had been predominantly closed for 11 months of each of the three years prior to sampling (i.e., since management was implemented). Since implementation, the areas had likely experienced low to moderate fishing pressure compared to open reefs [56]. Given these harvesting levels, the closure period may have been insufficient to lead to significant growth recovery of fish. Extending the duration of the closure, while maintaining the same levels of fishing pressure, may allow for greater growth gains. Even slightly longer fish can benefit fishers substantially in terms of yield (e.g., *L. rufolineatus* taken from periodically-harvested closures were on average 40% heavier than those from open reefs). In addition to the direct benefits of harvesting larger fish, there may also be secondary benefits (such as enhanced reproductive output) from the short term protection of larger fish, particularly when periods of closure are longer (e.g., [69]).

Across the Indo-Pacific, the most commonly reported use of periodically-harvested closures is for trochus fisheries management [36]. Trochus is arguably the most important commercially harvested marine product contributing to livelihoods of rural communities in the Indo-Pacific [70], however there are concerns about overexploitation of stocks throughout the region [71]. Periodically-harvested closures are perceived in some cases as a successful strategy for managing trochus fisheries due to observable recoveries during closure [65], and a history of stable catches in some areas where the strategy is employed [72]. As a fast growing, sedentary invertebrate, trochus would be expected to be suited to management by periodic harvesting strategies [32], [33]. However, if adult populations have been substantially depleted by periodic harvests, relatively long-term closures (2–3 years) may be required for cryptic juveniles to emerge and new recruits to settle [73], [74]. The decline in gleaning CPUE we observed, alongside anecdotal reports from fishers, suggests that the pressure on trochus stocks during periodic harvests can be intense. Harvesting intensity may be elevated because fishers are taking advantage of the window of opportunity to harvest this valuable commodity. The significantly smaller trochus caught from periodically-harvested closures may reflect the impacts of previous harvests and removal of larger (legal) size classes. Although unlikely in wild populations and for relatively short closure periods, there is also evidence that at high stocking densities, trochus shell growth is inhibited [71], [75]. While the mechanism leading to small sized trochus requires further investigation, these harvests have negative implications for fishers as small trochus yield less meat for human consumption, and small shells will fetch a lower market price [76]. The combination of enforced size limits, harvest quotas and periodic harvesting strategies has been shown to vastly improve trochus yield over the long term [50], [65]. This again reinforces that periodically-harvested closures may be more likely to achieve long-term fisheries objectives when restrictions on periodic harvests are concurrently applied.

## Conclusion

Periodically-harvested closures are amendable to local implementation and are frequently employed by co-management across the Indo-Pacific region. Although widely used as a management measure for multi-species fisheries, there have been few studies of their fisheries outcomes. We find that for multi-species fisheries, periodically-harvested closures may bolster catch rates of invertebrates, and lead to catches with slightly larger mean sizes for some finfish species, thereby meeting with some short-term community goals. Although effort during multi-species periodic harvests was much higher than for continuously fished reefs, conclusive evidence of short-term depletion was only found for invertebrate stocks, which are more likely than longer-lived fish to rebuild during closure periods. For long-term fisheries objectives, it is important to consider that in their current form, periodically-harvested closures in the Indo-Pacific; (1) may be applied in isolation to other effective resource-use regulations, (2) may only benefit taxa of high rebound potential, unless overall fishing pressure is substantially reduced, and (3) may result in elevated catch rates, increased abundance or larger fish, but these are realised only in a small proportion of fishing grounds and may be quickly reduced by intense pulses of fishing. The variability and flexibility of cycles of harvesting and closure applied in practice provide a mechanism for management to account for varying life history traits of target taxa, and to account for changed ecological conditions. However, this flexibility may potentially leave fisheries vulnerable to high levels of depletion when demand for marine resources is high. Data-intense or prescriptive management measures are poorly suited to co-management in developing countries. However, there is a need to complement local ecological knowledge with appropriate forms of monitoring and new knowledge generation to reassess, readjust and regulate periodic harvests. As demands for marine resources intensify, the application of complementary management measures in adjacent fishing grounds will become increasingly important for the long-term sustainability of small-scale fisheries.

## References

[1] Pauly D (2006) Major trends in small-scale marine fisheries, with emphasis on developing countries, and some implications for the social sciences. Maritime Studies (MAST) 4: 7–22.

[2] Foale SJ, Manele B (2004) Social and political barriers to the use of Marine Protected Areas for conservation and fishery management in Melanesia. Asia Pacific Viewpoint 45: 373–386.

[3] Christie P (2004) Marine protected areas as biological successes and social failures in southeast Asia. In: Shipley JB, editor. Aquatic Protected Areas as Fisheries Management Tools. pp. 155–164.

[4] Johannes RE (1978) Traditional marine conservation methods in Oceania and their demise. Annual Review of Ecology and Systematics 9: 349–364.

[5] Carrier JG (1987) Marine tenure and conservation in Papua New Guinea: problems in interpretation. In: McCay BJ, Acheson JM, editors. The Question of the Commons: The Culture and Ecology of Communal Resources. Tucson: The University of Arizona Press. pp. 142–167.

[6] Govan H (2009) Achieving the potential of locally managed marine areas in the South Pacific. SPC Traditional Marine Resource Management and Knowledge Information Bulletin 25: 16–25.

[7] McLeod E, Szuster B, Salm R (2009) Sasi and marine conservation in Raja Ampat, Indonesia. Coastal Management 37: 656–676.

[8] Hart DR (2003) Yield- and biomass-per-recruit analysis for rotational fisheries, with an application to the Atlantic sea scallop (*Placopecten magellanicus*). Fishery Bulletin 101: 44–57.

[9] Valderrama D, Anderson JL (2007) Improving utilization of the Atlantic sea scallop resource: An analysis of rotational management of fishing grounds. Land Economics 83: 86–103.

[10] Caddy JF, Seijo JC (1998) Application of a spatial model to explore rotating harvest strategies for sedentary species. Canadian Special Publication of Fisheries and Aquatic Sciences 125: 359–365.

[11] Sluczanowski PR (1984) A management-oriented model of an abalone fishery whose substocks are subject to pulse fishing. Canadian Journal of Fisheries and Aquatic Sciences 41: 1008–1014.

[12] Gendron L, Brethes JC (2002) Simulations of the impact of different temporal and spatial allocations of fishing effort on fishing mortality in a lobster (*Homarus americanus*) fishery. Canadian Journal of Fisheries and Aquatic Sciences 59: 899–909.

[13] Botsford LW, Quinn JF, Wing SR, Brittnacher JG (1993) Rotating spatial harvest of a benthic invertebrate, the Red Sea urchin, *Strongylocentrotus franciscanus*. In: Kruse G, Eggers DM, Marasco RJ, Pautzke C, Quinn TJ, II, editors. pp. 409–428.

[14] Pfister CA, Bradbury A (1996) Harvesting red sea urchins: Recent effects and future predictions. Ecological Applications 6: 298–310.

[15] Caddy JF (1993) Background concepts for a rotating harvesting strategy with particular reference to the mediterranean red coral, *Corallium rubrum* . Marine Fisheries Review 5: 10–18.

[16] Myers RA, Fuller SD, Kehler DG (2000) A fisheries management strategy robust to ignorance: rotational harvest in the presence of indirect fishing mortality. Canadian Journal of Fisheries and Aquatic Sciences 57: 2357–2362.

[17] Game ET, Bode M, McDonald-Madden E, Grantham HS, Possingham HP (2009) Dynamic marine protected areas can improve the resilience of coral reef systems. Ecology Letters 12: 1336–1346. 19807775 10.1111/j.1461-0248.2009.01384.x

[18] Kaplan DM, Hart DR, Botsford LW (2010) Rotating spatial harvests and fishing effort displacement: a comment on Game, et al. (2009). Ecology Letters 13: E10–E12. 20636770 10.1111/j.1461-0248.2010.01499.x

[19] Williams ID, Walsh WJ, Miyasaka A, Friedlander AM (2006) Effects of rotational closure on coral reef fishes in Waikiki-Diamond Head Fishery Management Area, Oahu, Hawaii. Marine Ecology Progress Series 310: 139–149.

[20] Jupiter SD, Weeks R, Jenkins AP, Egli DP, Cakacaka A (2012) Effects of a single intensive harvest event on fish populations inside a customary marine closure. Coral Reefs 31: 321–334.

[21] Cinner JE, Marnane MJ, McClanahan TR (2005) Conservation and community benefits from traditional coral reef management at Ahus Island, Papua New Guinea. Conservation Biology 19: 1714–1723.

[22] Cinner J, Marnane MJ, McClanahan TR, Almany GR (2006) Periodic closures as adaptive coral reef management in the Indo-Pacific. Ecology and Society 11: 31.

[23] Bartlett CY, Manua C, Cinner J, Sutton S, Jimmy R, et al (2009) Comparison of outcomes of permanently closed and periodically harvested coral reef reserves. Conservation Biology 23: 1475–1485. 19624531 10.1111/j.1523-1739.2009.01293.x

[24] Abesamis RA, Russ GR (2005) Density-dependent spillover from a marine reserve: Long-term evidence. Ecological Applications 15: 1798–1812.

[25] Russ GR, Alcala AC (2004) Marine reserves: long-term protection is required for full recovery of predatory fish populations. Oecologia 138: 622–627. 14716555 10.1007/s00442-003-1456-4

[26] Hilborn R, Stokes K, Maguire JJ, Smith T, Botsford LW, et al (2004) When can marine reserves improve fisheries management? Ocean & Coastal Management 47: 197–205.

[27] Russ GR, Stockwell B, Alcala AC (2005) Inferring versus measuring rates of recovery in no-take marine reserves. Marine Ecology - Progress Series 292: 1–12.

[28] Jennings S (2001) Patterns and prediction of population recovery in marine reserves. Reviews in Fish Biology & Fisheries 10: 209–231.

[29] Halpern BS, Warner RR (2002) Marine reserves have rapid and lasting effects. Ecology Letters 5: 361–366.

[30] Roberts CM, Bohnsack JA, Gell F, Hawkins JP, Goodridge R (2001) Effects of marine reserves on adjacent fisheries. Science 294: 1920–1923. 11729316 10.1126/science.294.5548.1920

[31] Russ GR, Alcala AC (2003) Marine reserves: Rates and patterns of recovery and decline of predatory fish, 1983–2000. Ecological Applications 13: 1553–1565.

[32] Jennings S, Reynolds JD, Polunin NVC (1999) Predicting the vulnerability of tropical reef fishes to exploitation with phylogenies and life histories. Conservation Biology 13: 1466–1475.

[33] Russ GR, Alcala AC (1998) Natural fishing experiments in marine reserves 1983–1993: roles of life history and fishing intensity in family responses. Coral Reefs 17: 399–416.

[34] Foale S, Cohen P, Januchowski-Hartley S, Wenger A, Macintyre M (2011) Tenure and taboos: origins and implications for fisheries in the Pacific. Fish and Fisheries 12: 357–369.

[35] Gelcich S, Hughes TP, Olsson P, Folke C, Defeo O, et al (2010) Navigating transformations in governance of Chilean marine coastal resources. Proceedings of the National Academy of Sciences of the United States of America 107: 16794–16799. 20837530 10.1073/pnas.1012021107 PMC2947917

[36] Cohen PJ, Foale SJ (2013) Sustaining small-scale fisheries with periodically harvested marine reserves. Marine Policy 37: 278–287.

[37] Feary DA, Cinner JE, Graham NAJ, Januchowski-Hartley FA (2011) Effects of customary marine closures on fish behavior, spear-fishing success, and underwater visual surveys. Conservation Biology 25: 341–349. 21129032 10.1111/j.1523-1739.2010.01613.x

[38] Bell JD, Kronen M, Vunisea A, Nash WJ, Keeble G, et al (2009) Planning the use of fish for food security in the Pacific. Marine Policy 33: 64–76.

[39] Foale S (1998) What's in a name? An analysis of the West Nggela (Solomon Islands) fish taxonomy. SPC Traditional Marine Resource Management and Knowledge Information Bulletin 9: 2–19.

[40] Froese R, Pauly D (2012) FishBase. World Wide Web electronic publication. Available: http://www.fishbase.org. Accessed August 2012.

[41] Choat JH, Axe LM (1996) Growth and longevity in acanthurid fishes an analysis of otolith increments. Marine Ecology-Progress Series 134: 15–26.

[42] Rasband WS (1997–2012) ImageJ. Bethesda: U.S. National Institutes of Health.

[43] Beverton RJ, Holt SJ (1957) 19: 533. (1957) On the dynamics of exploited fish populations: Great Britain Ministry of Agriculture, Fisheries and Food. 533 p.

[44] Harley SJ, Myers RA, Dunn A (2001) Is catch-per-unit-effort proportional to abundance? Canadian Journal of Fisheries and Aquatic Sciences 58: 1760–1772.

[45] Maunder MN, Sibert JR, Fonteneau A, Hampton J, Kleiber P, et al (2006) Interpreting catch per unit effort data to assess the status of individual stocks and communities. ICES Journal of Marine Science 63: 1373–1385.

[46] Pinheiro JC, Bates DM (2000) Mixed-eVects models in S and S-Plus. New York: Springer.

[47] Clarke K, Gorley R (2006) PRIMER v6: User Manual/Tutorial. Plymouth: PRIMER E. 192 p.

[48] Clarke KR, Warwick RM (2001) Change in marine communities:an approach to statistical analysis and intepretation Plymouth: PRIMER-E.

[49] Palomares MLD, Pauly D (2012) SeaLifeBase. Available: www.sealifebase.org. Accessed August 2012.

[50] Nash W, Adams T, Tuara P, Terekia O, Munro D, et al.. (1995) The Aitutaki Trochus Fishery: A Case Study. Noumea: South Pacific Commission. 72 p.

[51] Johannes RE (2002) The renaissance of community-based marine resource management in Oceania. Annual Review of Ecology and Systematics 33: 317–340.

[52] Thorburn CC (2000) Changing customary marine resource management practice and institutions: The case of Sasi Lola in the Kei Islands, Indonesia. World Development 28: 1461–1479.

[53] Allee WC, Park O, Emerson AE, Park T, Schmidt KP (1949) Principles of Animal Ecology. Philadelphia: W.B. Saunders Company. 837 p.

[54] Stoner AW, Ray-Culp M (2000) Evidence for Allee effects in an over-harvested marine gastropod: density-dependent mating and egg production. Marine Ecology Progress Series 202: 297–302.

[55] Bell JD, Purcell SW, Nash WJ (2008) Restoring small-scale fisheries for tropical sea-cucumbers. Ocean & Coastal Management 51: 589–593.

[56] Cohen P, Cinner J, Foale S (2013) Fishing dynamics associated with periodically-harvested marine closures. Global Environmental Change In press.

[57] Januchowski-Hartley FA, Graham NAJ, Feary DA, Morove T, Cinner JE (2011) Fear of Fishers: Human Predation Explains Behavioral Changes in Coral Reef Fishes. PLoS ONE 6. 10.1371/journal.pone.0022761 PMC3154266 21853046

[58] Januchowski-Hartley FA, Graham NAJ, Cinner JE, Russ GR (2013) Spillover of fish naivete from marine reserves. Ecology Letters 16: 191–197. 23126388 10.1111/ele.12028

[59] Murawski SA, Wigley SE, Fogarty MJ, Rago PJ, Mountain DG (2005) Effort distribution and catch patterns adjacent to temperate MPAs. ICES Journal of Marine Science 62: 1150–1167.

[60] Gutierrez NL, Hilborn R, Defeo O (2011) Leadership, social capital and incentives promote successful fisheries. Nature (London) 470: 386–389. 21209616 10.1038/nature09689

[61] Kulbicki M, Sarramegna S, Letourneur Y, Wantiez L, Galzin R, et al (2007) Opening of an MPA to fishing: Natural variations in the structure of a coral reef fish assemblage obscure changes due to fishing. Journal of Experimental Marine Biology and Ecology 353: 145–163.

[62] McClanahan TR, Cinner JE (2008) A framework for adaptive gear and ecosystem-based management in the artisanal coral reef fishery of Papua New Guinea. Aquatic Conservation-Marine and Freshwater Ecosystems 18: 493–507.

[63] Foale S, Adhuri D, Aliño P, Allison E, Andrew N, et al (2013) Food security and the Coral Triangle Initiative. Marine Policy 38: 174–183.

[64] Foale SJ (1998) Assessment and management of the trochus fishery at West Nggela, Solomon Islands: an interdisciplinary approach. Ocean and Coastal Management 40: 187–205.

[65] Foale S, Day R (1997) Stock assessment of trochus (Trochus niloticus) (Gastropoda : Trochidae) fisheries at West Nggela, Solomon Islands. Fisheries Research 33: 1–16.

[66] McCallum HI (1988) Pulse fishing may be superior to selective fishing. Mathematical Biosciences 89: 177–181.

[67] Dulvy NK, Polunin NVC, Mill AC, Graham NAJ (2004) Size structural change in lightly exploited coral reef fish communities: evidence for weak indirect effects. Canadian Journal of Fisheries and Aquatic Sciences 61: 466–475.

[68] Jennings S, Greenstreet SPR, Reynolds JD (1999) Structural change in an exploited fish community: a consequence of differential fishing effects on species with contrasting life histories. Journal of Animal Ecology 68: 617–627.

[69] Devlaming V, Grossman GD, Chapman F (1982) On the use of the Gonosomatic index. Comparative Biochemistry and Physiology a-Physiology 73: 31–39.

[70] FAO (2010) Marine fishery resources of the Pacific Islands Rome 58.

[71] Clarke PJ, Komatsu T, Bell JD, Lasi F, Oengpepa CP, et al (2003) Combined culture of Trochus niloticus and giant clams (Tridacnidae): benefits for restocking and farming. Aquaculture 215: 123–144.

[72] Evans SM, Gill ME, Retraubun ASW, Abrahamz J, Dangeubun J (1997) Traditional management practices and the conservation of the gastropod (Trochus niloticus) and fish stocks in the Maluku Province (eastern Indonesia). Fisheries Research (Amsterdam) 31: 83–91.

[73] Nash WJ (1993) Trochus. In: Wright A, Hill L, editors. Nearshore Marine Resources of the South Pacific. Suva: Institute of Pacific Studies, Forum Fisheries Agency, International Centre for Ocean Development. pp. 710.

[74] Lincoln-Smith MP, Pitt KA, Bell JD, Mapstone BD (2006) Using impact assessment methods to determine the effects of a marine reserve on abundances and sizes of valuable tropical invertebrates. Canadian Journal of Fisheries and Aquatic Sciences 63: 1251–1266.

[75] Amos MJ, Purcell SW (2003) Evaluation of strategies for intermediate culture of Trochus niloticus (Gastropoda) in sea cages for restocking. Aquaculture 218: 235–249.

[76] Richards AH, Bell LJ, Bell JD (1994) Inshore fisheries resources of Solomon Islands. Marine Pollution Bulletin 29: 90–98.

